# Incidence of mandibular osteoradionecrosis (MORN) after intensity modulated radiotherapy (IMRT) versus 3D conformal radiotherapy (3D-CRT): A systematic review

**DOI:** 10.4317/medoral.25459

**Published:** 2022-10-16

**Authors:** Carlos Arturo Céspedes-Ajún, Sara Amghar-Maach, Cosme Gay-Escoda

**Affiliations:** 1 Fellow in Master's Degree Program in Oral Surgery [EFHRE International University/FUCSO], Barcelona, Spain; 2 Fellow in Master's Degree of Oral Surgery and Implantology. [Faculty of Medicine and Health Sciences, Dental School, University of Barcelona] Barcelona, Spain; 3 Chairman and Professor of Oral and Maxillofacial Surgery, Faculty of Medicine and Health Sciences. Dental School University of Barcelona. Director of the Master's Degree Program in Oral Surgery and Implantology EFHRE International University/FUCSO. Coordinator/Researcher of the IDIBELL Institute. Head of the Oral Surgery, Implantology and Maxillofacial Surgery Department of the Teknon Medical Centre, Barcelona, Spain

## Abstract

**Background:**

Analyze the incidence of MORN after head and neck radiotherapy by two novel irradiation techniques, 3DCRT and IMRT and compare the success rates of distinct authors.

**Material and Methods:**

An electronic search in Pubmed (MEDLINE), Ovid, Google Scholar and Cochrane Library (Wiley), databases was conducted with the key words "Radiotherapy, Conformal"[Mesh] OR "Radiotherapy, Intensity-Modulated"[Mesh]) AND "Osteoradionecrosis"[Mesh] for all databases. The inclusion criteria randomized controlled trials (RCT), as well as prospective and retrospective cohort studies published in English; MORN patients treated with 3D-CRT y IMRT.

**Results:**

27 articles were selected from 194 initially found. 14 articles out of 27 were excluded and finally included 8 publications were included in the systematic review that were ranked according to their level of scientific evidence using the SORT criteria.

**Conclusions:**

When both RT techniques were compared; IMRT revealed a lower risk incidence of MORN development and enhanced dose constraint than 3D-CRT (less than 10%), this improvement could translate into less complications post RT treatment.

** Key words:**Radiotherapy, conformal, intensity-modulated, osteoradionecrosis.

## Introduction

Mandibular osteoradionecrosis (MORN) is defined as a zone of exposed necrotic bone in a previously irradiated area that fails to heal over a period of 3–6 months (1-5). According several authors (1-2) the etiopathogenesis of this disease lies in vascular and bone tissue damage that causes a hypoxic, hypocellular and hypovascular environment. For this reason, the lower jaw seems to be especially predisposed to the development of the disease (1,2,4-6), since blood supply is restricted to a lone functional terminal artery. The facial artery does not happen to be able to deliver enough blood vessels to make up for the loss of blood supply to the lower jaw afterwards fibrosis of the inferior alveolar artery happens (4,6). According to Maeschalck *et al*. (2) the determining factor in the development of MORN lies not only on alterations in regulation but also in the activation of fibroblastic action. It seems that the combination of non-replicating agonizing osteoblasts, together with an excessive proliferation of myofibroblasts, results in a decrease of bone structure, subsequently leading to the origin of vulnerable atrophic fibrous tissue on the irradiated area. According to some authors (4), the disease is considered mainly as a healing impairment, even though it could be associated with secondary infection. While the development of MORN because of decreased blood supply, and high radiation dose treatments (>60–75Gy); have been strongly suggested as a convincing etiological cause, its pathogenesis is noticeably multifactorial (1-8).

The local risk factors described by Moon *et al*. (1) include: tumor staging, site and bone proximity, as well as radiation field, dose systemic factors and risk behaviors such as smoking and alcohol consumption. However, poor oral hygiene and local trauma (extractions, etc.) before or after radiotherapy (RT) have been considered throughout history as the most important risk factors (1-8). MORN can be difficult to handle without the necessary knowledge for its management after diagnose. On the other hand, a good cancer treatment assessment and planning is of vital significance, as such it is important to diminish these potential risk factors to help prevent its development (6).

The reported incidence of MORN has decreased in recent years 20%, on the early 1940s and about 3-8% after late 90s (2000s) (1-5). This significant decline may be attributed to several aspects such as improvements in RT methods, which contrasting to conventional RT, can produce highly conformal dose distributions that result in a toxicity decrease and faster treatment periods. These dosimetric differences, in turn, can be theoretically translated into clinically relevant therapeutic advantages and possible enhancements in the identification and ease of MORN development factors (1-8). ]. The application of modern irradiation techniques as conformal radiation therapy (3D- CRT) or intensity-modulated radiation therapy (IMRT) allows a more distinct preservation of adjacent primary tumor tissues and a more precise assessment of high-irradiation and high-risk areas (1-4) which leads to a reduction in oral adverse effects, such as the risk of development of MORN.

The aim of this study was to perform a systematic review about the incidence of MORN after head and neck radiotherapy by two novel irradiation techniques: 3D-CRT y IMRT.

## Material and Methods

This article follows the Preferred Reporting Items for Systematic reviews and Meta-Analyses (PRISMA) statement (9).

- Eligibility criteria

A protocol that defined inclusion criteria, search strategy and outcomes of interest was developed. In order to select the appropriate literature, a PICO question was designed: What is the incidence of mandibular osteoradionecrosis in head and neck cancer patients treated with 3D conformal radiotherapy (3DCRT) vs. modulated intensity radiotherapy (IMRT Study inclusion criteria were (i) design: randomized controlled trials (RCT), as well as prospective and retrospective cohort studies published in English; (ii) population: MORN in head and neck cancer patients; (iii) intervention: patients treated with 3D-CRT or IMRT.

Presence or diagnosis of MORN of the jaws was the main outcome variable. Secondary explanatory variables such as radiation dose (RD), disease onset (DO), jaw location, and the descriptive variable follow-up time were registered.

- Search strategy

An electronic search in Pubmed (MEDLINE), Ovid, Google Scholar and Cochrane Library (Wiley), databases was conducted without restriction in year publication until September 13 2021.

The search strategy was ("Radiotherapy, Conformal"[Mesh] OR "Radiotherapy, Intensity-Modulated"[Mesh]) AND "Osteoradionecrosis"[Mesh] for all databases.

- Study selection and data selection process

The eligibility assessment was performed by first screening the titles and abstracts, or the whole text if sufficient information could not be gleaned from the abstracts to determine their eligibility. The irrelevant articles, or those who did not meet the eligibility criteria were discarded (Table 1). Next, the added value of high-quality articles was evaluated using the Strength of Recommendation Taxonomy Criteria (SORT)(10) as shown in Table 2.

**Table 1:**
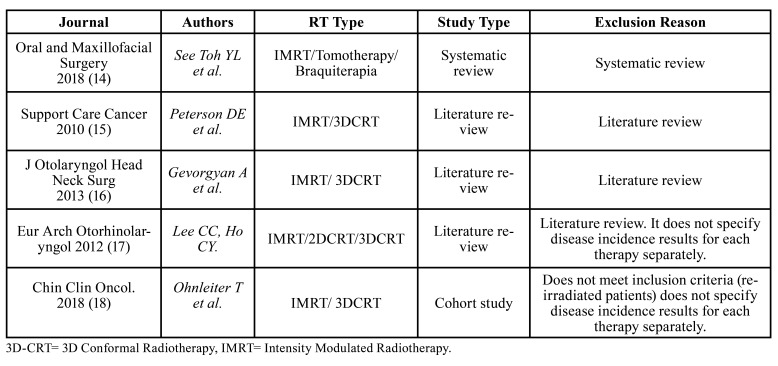
Discarded articles and reasons for exclusion.

- Risk of bias

Risk of bias was assessed according to the Newcastle-Ottawa scale guidelines (11). The quality of the studies was rated as good if they scored 3 to 4 stars in selection, 1 or 2 stars in comparability, and 2 or 3 stars in outcome sections. A fair quality score required 2 stars for selection, 1 or 2 stars for comparability, and 2 or 3 stars for outcome section; finally, a poor or deficient result was considered as one that displayed 0 to 1 star in selection, 0 stars in comparability, or 0-1 stars in the outcome section (Table 3).

- Statistical analysis

Statistical analyses evaluation was performed using Excel 2010 (Microsoft, Redmond, WA, US). 95% confidence intervals were reported for t test analysis. Comparison of categorical variables was achieved using Chi-square test (12).

## Results

-Study selection and description

**Figure 1:**
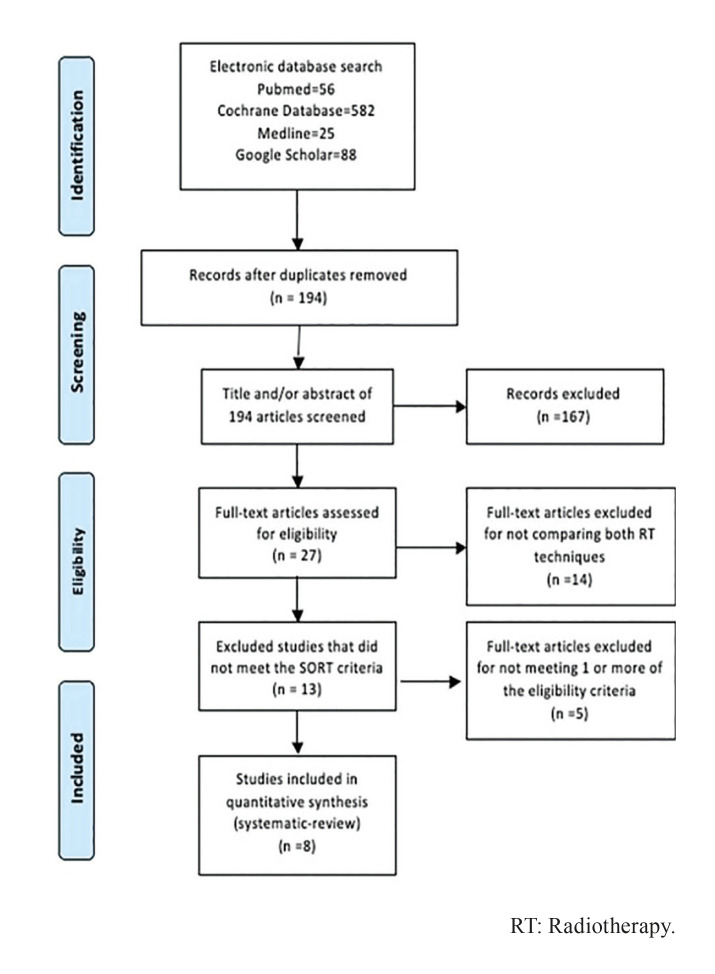
Flowchart of the review process, modified from the PRISMA statement.

The electronic database search, last updated on September 2021, yielded 751 articles. From the 751 records selected only 194 were considered significant to the subject. The abstracts of these 194 articles were then assessed for eligibility, leading to the selection of 27 full-text articles. Of the 27 relevant publications, only 13 articles were selected in which both RT techniques were compared and which met SORT criteria for proper reporting (10), and consequently endured further quality assessment methods and then incorporated in the systematic review. The remaining 14 publications did not meet the inclusion criteria and were therefore discarded. The flow chart for study selection tailored from PRISMA statement is exposed in Fig. 1 (9).

**Table 2:**
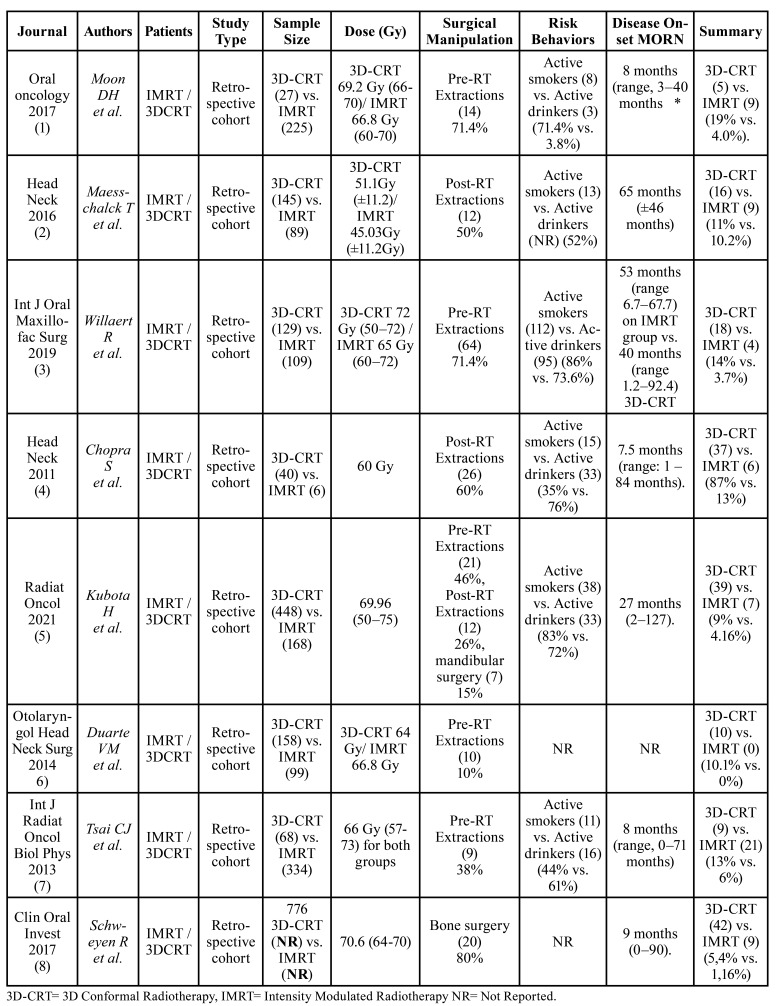
Articles included.

**Table 3:**
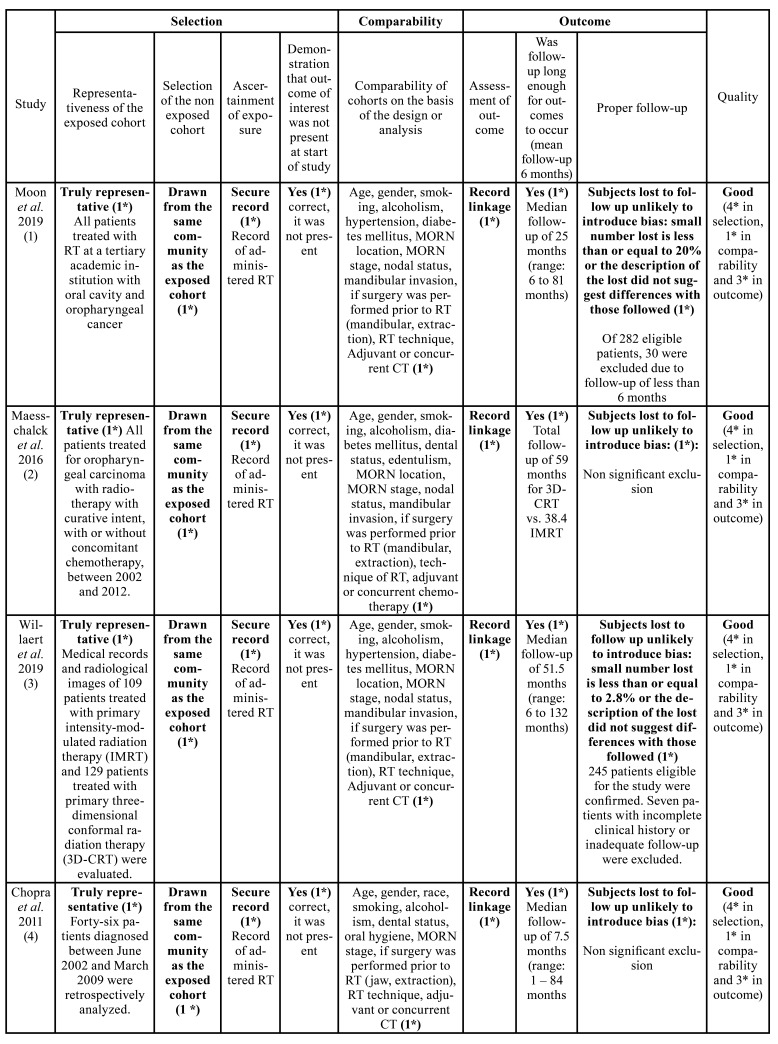
Risk of bias assessment (Newcastle–Ottawa Quality Assessment Scale criteria).

**Table 3 cont.**
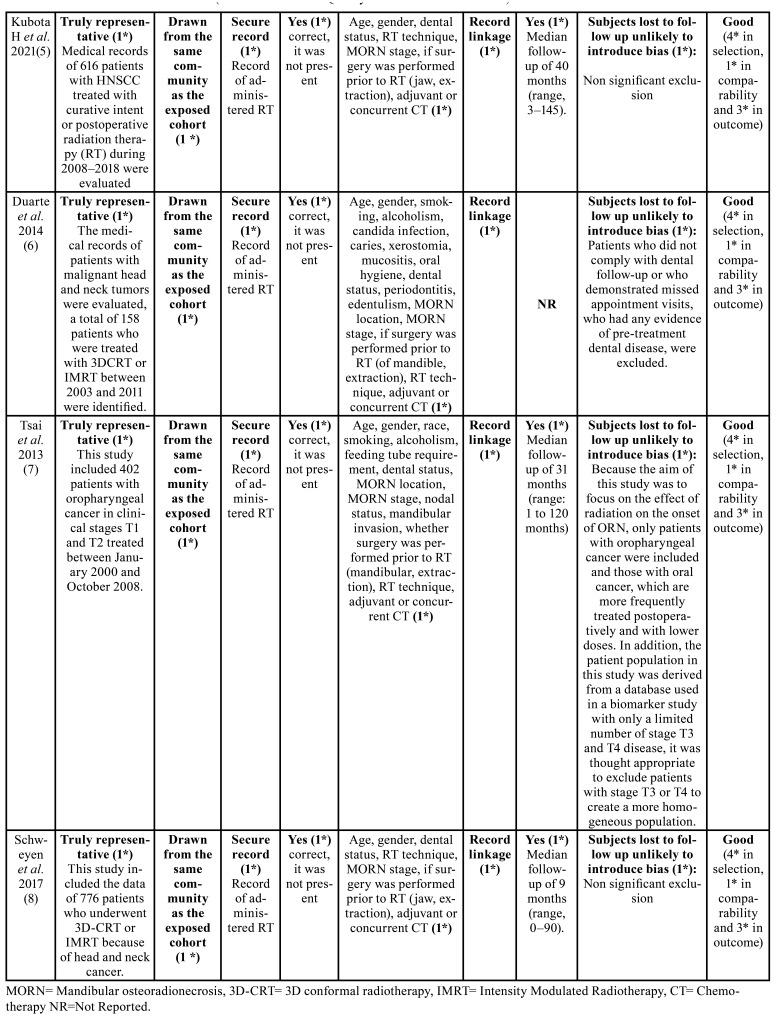
Risk of bias assessment (Newcastle–Ottawa Quality Assessment Scale criteria).

Of the 13 full-text articles that compared both RT techniques, only 8 were classified as level 2. The other 5 reports did not fulfill the standards to be considered with accepTable internal validity and were classified as level 3 and omitted from consequent examination. Although Kuhnt *et al*. publication (13) was not a duplicate article, it presented the same data as the research carried out by Schweyen *et al*. (8) and was not included in the systematic-review. Of the 5 studies discarded, the article by See Toh *et al*. was a systematic review (14), 3 were literature reviews (15-17), and one cohort study (18) that did not specify the disease incidence results for each RT technique, added to this, it included re-irradiated patients within the study population, which is an important variable that would directly affect the onset of the disease in treated patients. Since no examination fulfilled the base standard to be considered as high quality, there were no level 1 investigations of the SORT criteria classification (Table 1). In this investigation, the selected body of evidence (level 2) is made up of studies that presented solid and reliable findings. Therefore, the strength of the recommendation was considered level B, in light of the fact that the set of studies analyzed were considered reliable but of restricted quality. In depth analysis of variables are portrayed in Table 1, Table 2, Table 3 and Table 4 (10). Accomplishing quality assessment is difficult due to the great heterogeneity of study designs involved as portrayed in the present review, which would conduct, to an incorrect combination of data. We considered a meta-analysis to be unsuiTable as a result of discrepancies in study designs, interventions, participants, settings and outcome measures. Potential biases like inconsistent follow-up time, small sample size and uneven dose administration make calculating a single summary assessment of disease onset effect potentially ambiguous. Therefore, the current systematic review does not enclose meta-analysis.

- Risk of bias assessment

As shown in Table 3, the eight cohort studies analyzed (1-8) were classified as high quality, since most of them obtained scores equal or greater than 7 on the Newcastle-Ottawa scale (11). Although subject exclusion reasons were reported in detail in each article, in the study by Maesschalck *et al*. (2) the authors decided to exclude patients without clinical manifestations of the disease addressed as “radiographic MORN”, while Duarte *et al*. (6) excluded those who received primary surgical treatment and referred evidence of dental disease prior treatment, which directly affected the results of this systematic review, onset development time data was not available as well.

- Data extraction: qualitative synthesis.

Our results showed superior sparing capability results in the IMRT group than 3D-CRT in all the compared studies (1-8); Moon *et al*. (1) revealed significantly lower rates of MORN in patients subjected to IMRT and 3D-CRT treatment (19.0% vs. 4.0%), Willaert *et al*. (3) (14.0% vs. 3.7%), Tsai *et al*. (7) (13.0% vs. 6.0%), Duarte *et al*. (6) (6.3% vs. 0%), and Kubota *et al* (5) (9% vs. 4.1%) respectively. Maesschalck *et al* (2) showed no significant differences between both groups 3D-CRT n= 16 (11.0%) vs. IMRT n = 9 (10.2%). The incidence of MORN from a total sample size of 2.045 patients, when both RT techniques were compared was 13.2% [11.1-15.3] (n= 134) for 3D-CRT and 5.4% [4.0-6.8] (n= 56) for the IMRT group; suggesting enhanced mandible toxicity results (1-7).

Statistically significant outcomes were perceived between groups in patients receiving high RT doses ≥60 Gy. In 7 of the 8 studies analyzed (7.6%) n= 216, regardless of which technique was performed, all subjects who developed MORN underwent RT treatment under mean doses of ≥60 Gy (1,3-8). Exposure data for each individual radiological technique was not available in the study by Schweyen *et al*. (8). On the other hand, Maesschalck *et al*. (2) registered more cases of disease onset in the 3D-CRT group when doses below 60 Gy were administered in a sample size of 25 patients (1.13%). A time interval from RT administration to disease onset was examined, and the mean onset time was determined to be 31.1 months. Onset development time was not available in one study (6).

In this study among the 241 cases of MORN, which developed after IMRT and 3D-CRT techniques from 2821 head and neck cancer patients screened in all 8 articles, only 1 case (0.4%) developed in the upper jaw (3), while de remaining cases (99.6 developed in the lower jaw (1-8). It seems that bone surgery and dental extractions, both prophylactic and post-RT, persist as determining factors for the development of MORN in most of the evaluated series, in 6 of the 8 publications analyzed [1-5.8], 4.5% of the subjects (n=126) perceived an increase in the development of the disease, after surgical manipulation, demonstrating incidence rates of 50% or higher. In the publication by Moon *et al*. (1) 71.4% of the subjects that received pre-RT extractions (n=10) developed MORN. In Maesschalck *et al*. series (2) 50% of the subjects (n=12) developed the disease, after performing post-RT extractions; similarly, Chopra *et al*. (4) reported incidence rates of MORN in patients undergoing dental extractions post-RT in 60% of cases (n=26). Willaert *et al*. (3) indicated rates of 64% (n=14), from the 14 affected patients, 10 received post-RT extractions and 4 prophylactic tooth extractions. In the Schweyen *et al*. study (8) 80% of the subjects (n=20) who developed MORN underwent bone surgery. By reducing the total number of the cohort of patients who endured this procedure during primary tumor excision surgery, the relative frequency of MORN decreased from 6.6 to 3.6%.

**Table 4:**
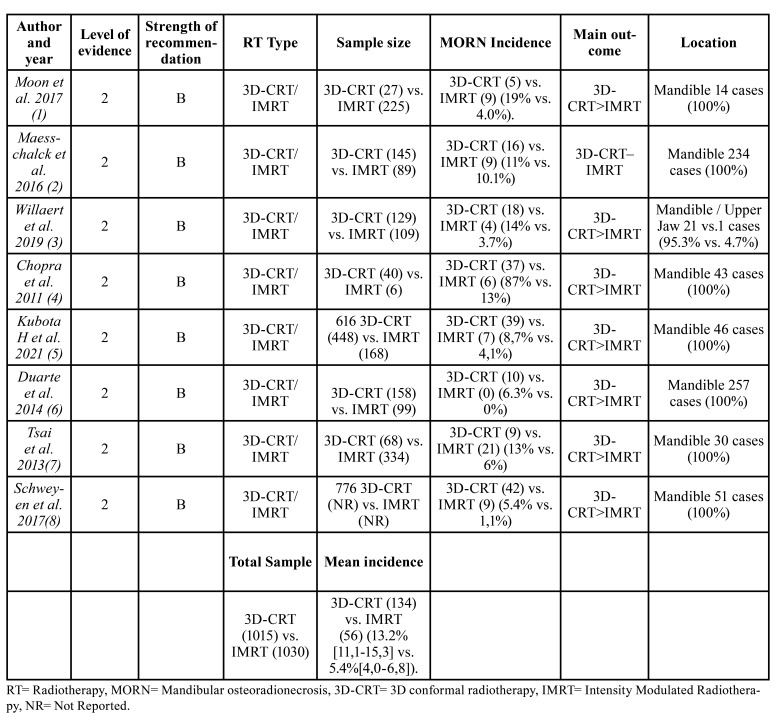
MORN incidence in comparative studies and SORT criteria application.

Kubota *et al*. (5) recorded the highest incidence of MORN, in this publication, 87% of the subjects exposed MORN (n=40) after receiving mandibular surgery pre-RT (n=7) and dental extractions both pre-RT (n= 21) as post-RT (n=12). The remaining 2 studies (6-7) indicated less than 40% rates in 1% of the population (n=19). In Tsai *et al*. publication (7) 38% of the subjects who developed the disease were treated with pre-RT extractions (n=9), while in the study performed by Duarte *et al*. (6) only 10% of the patients who received prophylactic extractions (n=10) developed the disease.

Risk behaviors were important factors in the development of the disease in 6 of the 8 studies (1-5,7). 7% of the subjects (n=197) reported being active smokers. In 3 of the 8 articles (2,3,5) more than 50% of the subjects who developed the disease disclosed regular smoking habits. In Maesschalck *et al*. research (2) 52% of subjects (n=13) developed MORN. Kubota *et al*. (5) reported rates of 83% in their series (n=38). Willaert *et al*. (3) reported the highest incidence levels of the disease in active smokers, 86% of the population (n=112). The remaining articles indicated incidence rates of MORN in less than 50% of subjects with regular smoking habits. Tsai *et al*. (7) reported MORN incidence rates in 44% of the investigated subjects (n=11), Chopra *et al*. (4) described disease 35% onset rates (n=15), on the other hand, Moon *et al*. (1) reported the lowest incidence levels of MORN in active smokers in 11% of the cases (n=8). The 2 remaining authors (6,8) did not report any related data referred to smoking and the development of MORN. Instead, 6.3% of the subjects (n=180) reported consuming alcohol regularly. In 4 of the 8 studies [3-5.7] more than 60% of the patients who developed the disease reported consuming alcohol regularly. Chopra *et al*. (4) indicated the highest incidence of the disease in alcohol consuming patients 76% (n=33), followed by Willaert *et al*. (3) with 73.6% of subjects (n=95), Kubota *et al*. (5) 72% (n=33) and Tsai *et al*. (7) with 61% of affected patients (n=16), while Moon *et al*. (1) portrayed the lowest incidence rate levels with 3.8% (n=3). The remaining 3 studies (2,6,8) did not report any data related to alcohol consumption and disease development.

## Discussion

The main purpose of the present study was to determine the incidence of MORN by means of novel irradiation techniques; based on the information analyzed in selected articles, the assumption we had was that patients treated with IMRT would ultimately manifest lower rates of MORN.

In our cohort of 2,045 patients, the MORN incidence was 13.2% [11.1-15.3] (n=134) for 3D-CRT and 5.4% [4.0-6.8] (n=56) for the IMRT group, patients treated with this last RT technique showed less than 5% rates, presenting consistent results with other modern series (1,3,5,6,8). Moon *et al*. (1) reported MORN incidences of 4% (n=9) of a 225 subject series with a mean follow-up of 25 months; Willaert *et al*. (3) reported 4 cases out of 109 patients with a mean follow-up of 51.5 months. Kubota *et al*. series (5) indicated incidences of 4% (n=7) in a population of 616 subjects, with a mean follow-up of 40 months. On the other hand Duarte *et al*. (6), achieved the lowest rates out of all examined studies, reporting 0 cases of MORN from a 99 patient sample treated with this technique. Schweyen *et al* (8). reported 9 affected patients from a 776 subject series, however, the number of patients undergoing each separate therapy was not provided, as explained in Table 1. In the 3 remaining studies (2,4,7) due to the significant distribution differences within groups undergoing each type of RT, the IMRT treated patients MORN rates ranged between 6% and 13%. However, after analyzing each article individually, it was concluded that the disease manifestation in each subject series was directly related not only to the different risk factors that triggered the disease, but also to the toxic habits of each patient, which will be discussed later. Tsai *et al*. (7) reported 6% (n=21) disease rates in a 334-subject population, with a mean follow-up of 8 months. Maesschalck *et al*. (2) on the other hand, reported 9 cases of MORN (10.2%) after a mean follow-up of 65 months from a sample of 89 patients, finally Chopra *et al*. (4) portrayed the highest disease rates of all the analyzed studies, reporting 13% frequencies (n=6) in a 6 subject series, with a mean follow-up of 7.5 months.

On the other hand, when analyzing the undergoing 3D-CRT RT patient series, the incidence MORN rates fluctuated between 5.4% and 87% (1-8), Kubota *et al*. patients series (5), reached disease incidences of 8.7% (n=39) of a 616 subject series with a mean follow-up of 40 months; the lowest incidence of MORN was found in the Schweyen *et al*. study (8) reporting 42 affected patients in a 776 subject series, within a 9 month mean follow-up time, however, this data is not conclusive because, as indicated before, the number of patients subjected to each separate therapy was not provided from the beginning. The rest of the examined articles (1-4,6-7) presented incidence rates greater than 10%. The Maesschalck *et al*. subject series (2) together with Duarte *et al*. (6) showed similar results 10.2% and 11% respectively. Likewise Willaert *et al*. (3) and Tsai *et al*. (7) exposed similar incidence rates 14% and 13% correspondingly. Moon *et al*. (1) like Chopra *et al*. (4) exhibited the highest disease rates. The first reported 5 MORN cases (19%) in a 27 subject series, with a mean follow-up of 8 months (1). While Chopra *et al*. (4) exposed the highest disease incidence rates, reporting 87% frequencies (n=37), with a mean follow-up of 7.5 months.

Throughout time a close association between maximum RT dosage and MORN onset (1-8), historically, MORN increased risk has been witnessed in patients receiving high dose levels of radiation (>60 Gy), in agreement with our study, most of the patients who developed osteoradionecrosis of the jaw underwent RT treatment under mean doses of ≥60 Gy (7.6%) n= 216 (1,3-8), in contrast Maesschalck *et al*. (2) reported more cases of MORN in the 3D-CRT group when doses under 60Gy were administered, 51.1 Gy vs. 45.03 Gy for the IMRT group, respectively. According to these authors, the main reason of the development of MORN lies in the dosimetry and bone volume distribution of the compromised irradiated area, regardless of the technique used (3DCRT or IMRT). Tsai *et al*. (7) support this theory and state that smaller mandibular volumes prone to receiving high doses may aggravate bone exposure caused by acute mucositis, therefore, a good pre-operative planning, in which the mandibular volumes exposed to high doses are limited, could help mitigate the appearance of MORN.

The lower jaw predilection promotes early literature evidence, which stated poor blood supply as the main reason for disease development, which in the past, Marx suggested in his theory of osteoradionecrosis pathophysiology by the triad hypoxia, hypocellularity and hypovascularity (19), in agreement with our findings in which the lower jaw persisted as the preferred location of MORN (99.5%) (1-8). In the study performed by Chopra *et al*. (4) post-RT secondary infection was significantly correlated with MORN appearance. According to these authors, secondary infections may be associated with the disease manifestation, although MORN arises mainly as a problem of ischemia and scarring, since strains of *Actinomyces* have been detected sporadically in the affected bone tissue.

Poor oral health, on the other hand, MORN progression has been widely accepted as an important risk factor, especially when post-RT extractions are performed on the lower wisdom teeth. According to the analyzed series (1-8), most of patients who required dental extractions were more likely to exhibit poor oral hygiene, smoke and to exhibit: comorbid conditions, poor bone quality, poor blood supply and therefore a increased risk of developing MORN. Bone surgery requirement, as well as tooth extraction before or after RT, persisted as determining factors in most cases (1-5,7,8). In the study performed by Moon *et al*. (1) 10 out of 14 patients who developed MORN (71.4%) underwent extractions prior to RT. In Maesschalck *et al*. series (2) post-RT tooth extraction remained as the most frequent disease trigger, in almost 50% of the patient population: 8 out of 16 in the 3D-CRT treated group and 4 out of 9 in the IMRT group. In the study by Chopra *et al*. (4) post-RT extraction history was predictive of MORN in 60% of the cases (n=26). In Willaert *et al*. series (3) the disease developed in 14 patients post-extraction sockets, however, in 4 out of 14 subjects, this followed after prophylactic extractions were performed. In the study by Tsai *et al*. (7) 37.5% of the subject population (n=9), developed MORN after experiencing post-RT extractions. In Kubota *et al*. series (5) MORN developed in 46% of the subjects (n=21) after pre-RT dental extractions were performed, in 26% after post-RT extractions (n=12); and in15% (n=7) after pre-RT mandibular surgery. However, in the study conducted by Duarte *et al*. (6) pre-RT tooth extractions did not show any significant outcomes, in this series, only 10.1% of the patients (n=10) developed the disease out of 28.3% of the subjects (n=28) who received dental extractions in the 3D-CRT group; in contrast, the patients who received extractions in the IMRT group 20.3% (n=12) did not expose any MORN cases. In the study by Schweyen *et al*. (8) 80% of the patients who manifested MORN (n=20) endured bone surgery. By reducing the total number of the cohort of patients who underwent this procedure during tumor surgery, the relative frequency of MORN would have diminished from 6.6 to 3.6%. According to these authors, the fact that 10 of these 20 patients were mandibular edentulous before RT treatment reinforces the relationship between bone surgery and disease progression risk.

Similarly, the risk behaviors of each patient were influential factors on the disease development in 6 of the 8 studies examined (1-5,7). In the study by Moon *et al*. (1) MORN rate was higher in current smokers than in non-smokers (11% vs. 3.4%). In these series, out of the 14 MORN subjects, 11% (n=8) were active smoker cases, 3.8% (n=3) were heavy drinkers and 6.3% (n=11) indicated light or no alcohol intake. In Tsai *et al*. series (7) out of the 30 MORN affected patients, 64% of the subjects (n=16), declared regular alcohol consumption, while 24% (n=6) indicated previous alcohol consumption, regarding smoking habits, 44% of the subjects (n=11) reported being active smokers and 32% (n=8) indicated having smoked in the past. Maesschalck *et al*. (2) also reported a high MORN incidence in active smokers, in 52% of the population (n=13). Willaert *et al*. (3) reported higher MORN rates in regular smoking patients, in these series of 129 subjects in the 3DCRT group, 86.8% (n=112) declared tobacco consumption and 73.6% (n=95) reported regular alcohol consumption, subsequently, 14% (n=18) developed the disease, in the IMRT group 109 subjects 86.2% (n=94) reported tobacco consumption and 73.4% (n=80) reported regular alcohol consumption, 3.7% (n=4) developed the disease later. Likewise, in Kubota *et al*. series (5) from 46 MORN affected patients, 83% (n=38) related being active smokers and 15% (n=7) did not report smoking habits; Regarding alcohol consumption, 72% (n=33) described past alcohol consuming history, while 24% (n=11) reported light or no alcohol intake. In contrast, in the Chopra *et al*. study (4) the risk behaviors did not play a significant role in the development of the disease; In this study, out of the 43 patients who developed MORN, 35% (n=15) reported smoking actively, while 65% (n=28) stated not having any smoking habits; however, 76% of the patients who developed the disease (n=33) consumed alcohol regularly and 24% (n=10) did not report alcohol intake. According to these authors, the continuous consumption of tobacco and alcohol has been related to the appearance of MORN, justified in terms of chronic ischemic damage, vasoconstriction and vasculitis of small caliber vessels.

In the present study, most of patients were treated with IMRT compared with 3D-CRT, and a trend towards less MORN rate was portrayed. It is important to emphasize the alikeness of results when estimating the incidence of IMRT between each group; the mean outcome incidence among all registered studies was 5,4% (1-7), demonstrating marked homogeneity in each analysis thus supporting consistent conclusions. However, the current results should be treated with discretion as significant limitations, one of the most important being the retrospective non-randomized nature of the study, which led to divergent incidence conclusions between publications. Going deeper into this point, it is important to highlight the great variability of results according to the inclusion criteria and evaluation of the disease in the analyzed publications (2,6). In the series by Maesschalck *et al*. (2) the authors decided to exclude the cases of "radiographic MORN", i.e. patients with intact mucosa and no clinical outcomes; while Duarte *et al*. (6) opted for the exclusion of patients with any evidence of pre-treatment oral pathology (e.g., caries, infection, periodontitis) as well as those who indicated extractions prior to dental extractions indicated prophylactically. By omitting these data from the total number of the population, the cohort tends to be heterogeneous, thus affecting the relative frequency of the disease, directly influencing the results of this study. According to Duarte *et al*. (6) a prospective randomized study would be a better evaluation to compare the incidence rates of MORN, however, it is still difficult to implement since currently IMRT therapy has been included as standard treatment due to its theoretical advantages.

Because the MORN development risk is a very fluctuating outcome, thus it may develop after several years post RT treatment, it is crucial an appropriate follow-up period in published studies when comparing or assessing data, to deliver effective results. Disease onset as expected was a very inconsistent variable among all studies (range: 7.5 - 65 months); this may be due to multiple aspects specific to each study (surgery performance, oral treatment, etc.) and of particular characteristics such as systemic factors and risk behaviors. Due to the retrospective condition of the present report, several limitations such as small sample size in some of the analyzed studies, as those exposed by Chopra *et al*. (4) (46 patients), inconsistent follow-up time as mentioned earlier and uneven dose administration in each study, these biases should be removed in further researches.

## Conclusions

In conclusion, when both RT techniques were compared; IMRT revealed a lower risk incidence of MORN development and enhanced dose constraint than 3D-CRT (less than 10%), this improvement could translate into less complications post RT treatment. However, the deficient number of quality articles comparing these two RT techniques makes it difficult to draw a concrete conclusion. It also should be pointed that not only the application of these new techniques but the reduction of risk factors, interdisciplinary patient supervision and correct monitoring is essential in the management of this affection. On the other hand, it is important to stand out that MORN alone is not a determining disease to conclude which RT technique is the best, other late toxicity effects such as xerostomia, trismus or dysphagia should be considered in future comparative studies. Due to the limitations of this review, as mentioned above, we recommend the implementation of more quality investigations in the future.
